# Combination adjunctive nebulized furosemide and salbutamol versus single agent therapy in COPD patients: A randomized controlled trial

**DOI:** 10.1016/j.amsu.2020.07.005

**Published:** 2020-07-18

**Authors:** Mohammadali Saba, Abdoulhossein Davoodabadi, Azin Ghaffari, Hamidreza Gilasi, Babak Haghpanah

**Affiliations:** a Department Pulmonary, Shahid Beheshti Hospital, Kashan, Iran; b Departments of Surgery, Kashan University of Medical Sciences, Kashan, Iran; c Internal Medicine, Shahid Beheshti Hospital, Kashan, Iran; d Departments of Epidemiology & Biostatistics, Kashan University of Medical Sciences, Kashan, Iran; e Orthopedic Surgery, Kashan University of Medical Sciences, Kashan, Iran

**Keywords:** COPD, Salbutamol, Furosemide, Combination therapy, Adjunctive effects, COPD, Chronic Obstructive Pulmonary Disease, mMRC, modified Medical Research Council, FEV1, forced expiratory volume/second, FVC, forced vital capacity

## Abstract

**Background:**

COPD patients often require multiple therapies to enhance their lung function and reduce their symptoms in exacerbations. This study aimed to investigate the relative effects of combination adjunctive nebulized furosemide and salbutamol therapy versus single agent treatment in COPD patients.

**Methods:**

Sixty-nine COPD patients were randomly divided into two groups. The first group (G1, 34 cases) received salbutamol in their first episode. The second group (G2, 35 cases) received furosemide in their first episode. Spirometry indices (FEV1, FVC, and FEV1/FVC), mMRC and BORG (COPD assessment) were assessed and recorded for all patients.

To study the efficacy of combination adjunctive therapy, in 2nd episodes, the nebulized furosemide was added to nebulized salbutamol in the G1, and nebulized salbutamol was added to nebulized furosemide in G2. The aforementioned indices were then re-assessed.

**Results:**

The mean age was (64.92 ± 11.71 years, 55% males. The use of nebulized furosemide and salbutamol as single agents slightly improved the spirometeric parameters, but it was not noteworthy compared to the significant improvement of the FEV1, FVC, FEV1/FVC, mMRC, and Borg parameters with combination therapy (*p*-value< 0.001). In the first episode, there was no difference in spirometeric indices, between groups (*p*-value > 0.1), so furosemide is considered as effective as nebulized salbutamol. Also, the results of sequential drugs administration, in the two groups was similar.

**Conclusion:**

Conjunction of nebulized furosemide and salbutamol is more effective than single therapy and can be considered as preferred drug regimen without any reported side effect in the treatment of COPD.

## Highlights

• Our study revealed.
• Nebulized furosemide is as effective as nebulized salbutamol in stable COPD treatment.
• Adding nebulized furosemide to nebulized salbutamol considerably improves thespirometry indices, helps to better management of COPD, without any side effect.

## 1 Introduction

### 1.1 Background and objectives

Chronic obstructive pulmonary disease (COPD) is a poorly reversible disease of the lungs, with significant morbidity and mortality [1,2]. The mortality rates of COPD patients during 1970–2002 have been doubled [3]. Severe dyspnea, as a dominant symptom, fatigue and disability, strongly predict impaired physical activity [4,5].

Patients with COPD has a high-risk of short and long-term death and postoperative infections after thoracic surgery. In patients undergoing surgery, impaired spirometry indices have been proved to be prognostic markers and such patients needs more COPD stabilization and careful monitoring of infection signs [6,7]. Reduction in the tolerance of physical activity, is the strongest predictor of mortality in COPD patients [8].

Despite high prevalence of COPD and implementation of many treatment guidelines, effective management has still remained a challenge in healthcare fields. Evidence shows that many of the patients do not received enough treatment [9].

Current management of COPD patients is to relieve dyspnea, minimize exacerbations, reducing ventilatory demand, improve exercise performance, and decrease mortality, which is achieved through prescription of a β2-agonists such as salbutamol [10,11]. Recently, studies suggest the possible effect of furosemide inhalation in COPD patients, and improvements in exertional dyspnea and exercise tolerance has been reported [12]. Nebulized furosemide has been shown to moderate the activity of sensory afferents in animals’ airways [13], and relieve sensation of experimentally induced dyspnea by various respiratory stimuli in healthy humans [14]. On the other hand, inhaled furosemide has many beneficial effects on the airway epithelium such as improvements in exercise induced asthma and inhibition of cough in asthmatics in healthy volunteers [[15], [16], [17], [18]].

Conjunction of furosemide with salbutamol (as a standard drug) may be an acceptable therapy and may have promising results in COPD management in future, however, the clinical evidence to support it as the standard therapy, is still insufficient [19]. This study is aimed to investigate the effect of combination adjunctive nebulized furosemide and salbutamol versus single agent therapy in COPD patients as a treatment protocol in two separate groups with more attention to their interactions and combination effects.

To the best of the authors’ knowledge, no study has assessed the adjunctive effects in pharmaceutical treatment of the stable COPD patients.

## 2 Patients and methodology

### 2.1 Trial design

This study was conducted on stable COPD patients, aged more than 40 years old, in a teaching Hospital from Oct 2018 to Dec 2019 (Research Registry UIN: 5605, institutional ethic committee registration# 1397.006) and WHO IRCTIRCT201707126187N5. The report is based in line with the CONSORT criteria [20].

All Spiro metric parameters were obtained by a pulmonologist using a mass flow spirometer (Ganshorn Medizin Electronic GmbH, Germany) and were measured according to American Thoracic Society recommendations and expressed as absolute values [21]. COPD intensity was primarily assessed by considering FEV1/FVC ratio as a fixed cut-off [22]. Then, these ratios were based on grouping the patients as stated by Global Initiative for Chronic Obstructive Lung Disease (GOLD) [23]. All patients were also evaluated by both modified Medical Research Council (mMRC) dyspnea scores and Borg scale. mMRC as a clinical scale of dyspnea estimates the severity of dyspnea in 5 grades (0–4). Our patients had dyspnea grade of 2–3 according to mMRC [24]. Borg scale was another tool to measure dyspnea severity and is rated numerically from 0 to 10 [25]. We categorized the scale for dyspnea at the same time of measuring the spirometry indices (FEV1, FVC, and FEV1/FVC). The patients in this study were in group 2, 3 of COPD according to GOLD.

### 2.2 Inclusion criteria

Stable COPD patients documented by the pulmonologist, (FEV1, FVC, (FEV1/FVC) less than 70%, irreversible response to inhaled β-agonist (FEV1increase <12% and <200 cc after15min from baseline), symptoms last about 5 years of breathlessness and, productive cough).

### 2.3 Exclusion criteria

Patients with history of other lung diseases such as pneumonia, idiopathic pulmonary fibrosis, consolidation, congestive heart failure, exacerbated, asthma or asthma-COPD overlap. Smoking more than 15 packs/year or, using furosemide and other diuretics also were excluded.

### 2.4 Sample size calculation

Determined for a one-way comparison of means (matched pairs *t*-test) based on a desired statistical power (1 − β) of 0.8 at a level of 0.05. The effect size used in the calculation (f = 0.3) was based on changes in FEV1 (L) in a prior study [15]). So the number of patients needed for our experiment was 68 cases.

### 2.5 Randomization

The patients were randomly divided in Group I and Group II, based on their reception number.

Nebulizer drug was delivered as aerosols. The patients and the physicians involved in examination and assessment of the patients were blind to the type of the drugs administered. We conducted a randomized double-blind, clinical trial in parallel groups for comparing the efficacy of nebulizer furosemide and nebulized salbutamol each alone and adjunctive therapy results unstable COPD patients.

### 2.6 Pere-intervention considerations

All patients were stable so they were instructed to withheld short-acting β2-agonists (4 h), short-acting anticholinergic (6 h), long-acting β2-agonists (12 h), long-acting anticholinergic (24 h), short-acting theophylline (24 h), and long-acting theophylline (48 h) before study enrollment.

### 2.7 Intervention

To evaluate the possible interaction between nebulized salbutamol and nebulized furosemide, two episodes were defined in each group; the 1st episode implicated the first drug administered alone and the 2nd episode indicated the effect of combination of the two drugs.

Salbutamol and furosemide were nebulized in doses of 5 mg and 40 mg, respectively, by means of a jet nebulizer (Pari, Sternberg, Germany). We ensured that none of the patients inhaled any bronchodilator or nebulized furosemide for a period 4 h.

Spirometry indices (FEV1, FVC, and FEV1/FVC), mMRC, BORG scale were measured before the beginning of the first nebulization as baseline for each group and then after the each administration of the drugs.

The First Group (G1, 34 cases) received one dose of nebulized salbutamol (5 mg) and the second group (G2, 35 cases) received furosemide (40 mg).

For assessment of combination adjunctive efficacy, in 2nd episode the nebulized furosemide was added to nebulized salbutamol in G1, and nebulized salbutamol was added to nebulized furosemide in G2.

**Fig. 1:**
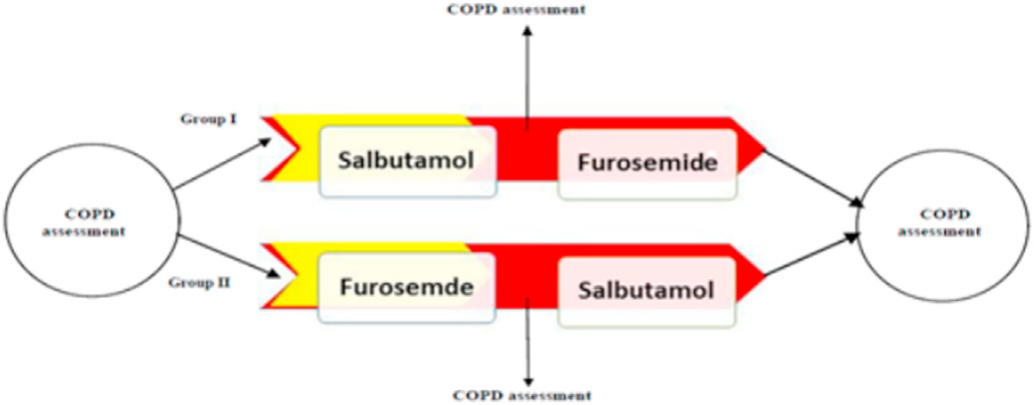
**Study design:** The yellow show the 1st and red arrows 2nd episodes. (For interpretation of the references to colour in this figure legend, the reader is referred to the Web version of this article.)

The study design is presented in Fig. 1.

The study was performed in out pulmonology ward where each patient was assessed by the same physician before and after interventions in the same day and with the same instruments. Physical activity was performed to assess the change in BORG or mMRC. According the mMRC grade before study we asked patients to do the activity at a level up to the level before study. BORG number was assessed as patients were exercised by activity question.

### 2.8 Data collection

Data collection was commenced after study approval and obtaining informed consent from all participants. During the study period, eligible cases consecutively were assessed in pulmonology ward each participant were subjected to measure the spirometry indices (FEV1, FVC, and FEV1/FVC) and examined mMRC, and Borg as base line and then after 1st and 2nd episode of nebulized drug administration.

### 2.9 Statistical analysis

A paired sample *t*-test was used to determine the difference between episodes within groups. This statistical procedure that is also called the dependent sample *t*-test is commonly used to evaluate the mean difference among two series of observation. The main application of this strategy is repeated measurement designs, namely at the end of each process or treatment method [26].

Moreover, a two-sample *t*-test was applied to compare two episodes between groups. This approach examines whether the differences in means between two independent populations is equal to a target value. An important general application of the method is to investigate if a new process or treatment option is superior to its current counterpart [26].

In this study, the results of each episode were compared with the next episode in a given group and then the two groups were compared with each other in terms of corresponding episodes. Therefore, the effects of the two drugs in addition to their individual effects could be distinguished. The statistical analysis of data was performed using Minitab v.26 (Minitab Inc., State College, PA, USA) and SPSS v. 23 (IBM, USA) software packages. All results were expressed in means and standard error and an alpha (α) at 95% confidence interval (*p*-value = 0.05) was considered for statistical significance.

### 2.10 Post-intervention considerations

Standard monitoring of groups was done, concomitant with closed clinical observation and pulse oximetrp after performing.

Physical activity, and continuing appropriate bronchodilator offered in each groups until the patients being discharged.

## 3 Results

Sixty-nine cases were enrolled in this study, mean age was (64.92 ± 11.71 years), 55(79.7%) of participants were male, demographics characteristics are summarized in Table 1.

**Table 1:** Demographics of the enrolled participants.

Groups	Frequency	Sex		Age (years)	
		Male	Female	Mean	SD
Group I	34	27	7	67.26	10.09
Group II	35	28	7	62.65	12.83
Total	69	55	14	64.92	11.71

In the both groups, there was no significant difference in sex an age distribution. The spirometric indices as well as the mMRC and Borg date in different episodes are presented in Table 2.

**Table 2:** Comparison of the drugs’ effects as pulmonary functional parameters within groups^a^.

Group	Index	Baseline	1st episode	2nd episode	*p*-value^b^	*p*-value^c^
Group I	FEV1 (L)	1.30 ± 0.58	1.33 ± 0.60	1.65 ± 0.76	0.30	0.001
	FVC (L)	2.20 ± 0.84	2.17 ± 0.88	2.63 ± 1.07	0.180	0.001
	EV1/FVC (%)	59.64 ± 16.00	60.68 ± 13.80	63.06 ± 14.4	0.175	0.019
	MMRC	2.35 ± 1.20	2.05 ± 1.20	0.79 ± 1.12	0.10	0.001
	Borg	7.38 ± 2.50	6.82 ± 2.34	3.56 ± 2.63	0.001	0.001
Group II	FEV1 (L)	1.38 ± 0.75	1.40 ± 0.75	1.77 ± 0.90	0.21	0.001
	FVC (L)	2.37 ± 1.10	2.31 ± 1.10	2.86 ± 1.32	0.041	0.001
	EV1/FVC (%)	57.65 ± 16.13	58.48 ± 15.94	3.06 ± 14.86	0.270	0.001
	MMRC	2.11 ± 1.15	2.00 ± 1.23	0.60 ± 1.06	0.103	0.001
	Borg	7.82 ± 2.47	7.40 ± 2.41	3.43 ± 2.21	0.001	0.001

a All values presented as means ± SD.

b Comparing baseline to the 1st episode.

c Comparing baseline to the 2nd episode.

The results showed that although all of the parameters improved in both groups after the first episode of the treatment, the improvement in pulmonary indices (FEV1, FVC, FEV1/FVC, and MMRC) was not significant (*p*-value > 0.05). Borg scale, however, had a significant improvement after the 1st episode (*p*-value < 0.001). (Table 2).

After the administration of second episode of the treatment, the FEV1, FVC, FEV1/FVC, MMRC, and Borg parameters, significantly improved in both groups compared to the baseline spirometry indices (*p*-value < 0.001). Table 2). The two groups were not significantly different in their response to their 1st and 2nd episode of the treatment (p-value > 0.1, Table 2).There was no significant difference in FEV1, FVC, FEV1/FVC, mMRC, and Borg parameter between each group (*p*-value > 0.1, Table 3). The sequence of the drug administration had no significant effect on the efficacy of the combination therapy (Table 3).

**Table 3:** A comparison of the drugs’ effects on pulmonary functional parameters between groups.

Index	Episodes	Group I (mean ± SD)	Group II (mean ± SD)	*p*-value^a^
FEV1 (L)	1st episodes	1.33 ± 0.60	1.40 ± 0.75	0.84
	2nd episodes	1.65 ± 0.76	1.77 ± 0.90	0.59
FVC (L)	1st episodes	2.17 ± 0.88	2.31 ± 1.10	0.56
	2nd episodes	2.63 ± 1.07	2.86 ± 1.32	0.56
FEV1/FVC	1st episodes	60.68 ± 13.80	58.48 ± 15.94	0.84
	2nd episodes	63.06 ± 14.45	63.06 ± 14.86	0.27
mMRC	1st episodes	2.00 ± 1.23	2.00 ± 1.23	0.16
	2nd episodes	0.79 ± 1.12	0.60 ± 1.06	0.87
Borg	1st episodes	6.82 ± 2.34	7.40 ± 2.41	0.45
	2nd episodes	3.56 ± 2.63	3.43 ± 2.21	0.28

a Independent sample T test for comparison of means between two groups.

### 3.1 Outcome

Since we selected the stable COPD patents as defined: patient who clinically is stable with no exacerbations or hospital admissions in the last 6 month, so the patents were in fairly good general condition, during the course of protocol was well, all of them were discharged from hospital with good condition.

## 4 Discussion

COPD patients are more susceptible to postoperative complication after thoracic surgery (8) stabilization helps them decrease the respiratory disability and physical activity-related breathlessness (6.7).

Conventional treatment options for COPD patients include administration of β2-agonist, anticholinergic, and glucocorticosteroid agents. More investigation is needed to define the optimal mode of pharmacotherapy in this group of patients [27,28]. Nebulized furosemide provides an additional therapeutic option to alleviate dyspnea and other physiologic respiratory parameters in COPD exacerbation.

There is increasing evidence suggesting that nebulized furosemide could be an option as single or combination therapy with Nebulized salbutamol. The effectiveness of nebulized furosemide in decreasing airflow obstruction in acute asthma exacerbation also has been reported [[29], [30], [31]].

Our study suggest that the result of administration of either drugs as single therapy was not significantly different in the terms of spirometric indices and mMRC and Borg results. So nebulized furosemide is as effective as nebulized salbutamol as a known standard drug for COPD treatment and can alleviate dyspnea and other physiologic respiratory parameters, without any cardiovascular comorbidity or arrhythmias which may be seen salbutamol [32]. We had considerable results with the use of combination therapy with significant improvement of FEV1, FVC, FEV1/FVC, MMRC, and Borg parameters in both groups when compared to the baseline (*p*-value < 0.001), there was no difference if either drug was added to the single therapy with other drug and the results in either case was similar.

The result of our study with a significant bronchodilatory effect in stable COPD is similar to a study by Hojat et al. that showed 40 mg nebulized furosemide as an adjunct to the conventional treatments improved significantly the FEV1, dyspnea, pH, mean blood pressure, and heart rate in patients with COPD exacerbation [33], another study with the same results was reported in children with mild asthma [34]. This study is line with several other studies which demonstrated that inhalation of nebulized furosemide (40 mg) compared with nebulized 0.9% saline decreased intensity ratings of breathlessness provoked by a variety of respiratory stimuli at rest in healthy adults or by constant-load cycle endurance exercise testing in COPD patient [[35], [36], [37], [38], [39]]. In a study by Ong KC et al., the effect of inhaled furosemide on dyspneic sensation during exercise testing with placebo has been investigated. They showed a significant improvement in mean FEV1 and FVC after inhalation of furosemide (p = 0.038 and 0.005, respectively) but not after placebo, and they conclude that inhalation of furosemide alleviates the sensation of dyspnea induced by constant-load exercise testing in patients with COPD and that there is significant bronchodilation after inhalation of furosemide compared with placebo in these patients [40].

In the above-mentioned study, it has been showed that nebulized furosemide have a positive influence on dyspnea. They assessed the superiority of nebulized furosemide plusplacebo versus nebulized salbutamol. In our study we assessed the effect of combination therapy with single therapy by sequentially adding one regimen to another.

Although the role of furosemide used as an adjunctive treatment for acute asthma and COPD exacerbation is an improving issue [[29], [30], [31]], some other studies have reported an inert effect for nebulized furosemide [[41], [42], [43]].

Despite extensive investigation, the mechanisms underlying relief of breathlessness with nebulized furosemide has not yet been understood. Several mechanisms have been suggested for anti-dyspneic action of nebulized furosemide including a direct protective impact on airways in addition to a protective effect against cholinergic, non-cholinergic, and non-adrenergic contraction of smooth muscles, the latter action providing an increased vascular response, improving micro vascular leakage to counteract evaporation of water, and vasodilatation [44,45]. In fact, the anti-asthmatic performance of furosemide is not only related to its effects as a diuretic agent, but also to the ability of dilating airway vasculature leading to increase of blood flow that supplying lung tissue [46].

The therapeutic effect of nebulized furosemide is more than its oral intake which leads to an increased diuresis via transmission of sodium, potassium, and chlorine ions in the ascending limb of the Henle loop [29]. Nebulized furosemide has a direct protective effect on the airway [47,48]. It even improves dyspnea in cancer patients [49]). Other mechanisms have been proposed in both animal and human studies e.g.prolonging the breath-holding time and alleviating respiratory discomfort in healthy volunteers [50], changing the activity of pulmonary stretch receptors (PSRs) which provide sensory feedback information on lung expansion via the vagus nerve to cortical and sub cortical regions of the brain which in turn may be implicated in the perception of breathlessness [51,52].

Our cases were stable COPD patients that may not resemble the general population, however, they were really the patients who were not in exacerbation, but had dyspnea in their daily activities.

Overall, these findings suggest that combination therapy can be a viable option in stable COPD patients. This combination therapy may have possible synergistic effect or adjunctive effect, yet optimizing the combination measure still needs to me more investigated in future.

## 5 Conclusion

Nebulized furosemide is as effective as nebulized salbutamol in stable COPD treatment. Adding nebulized furosemide to nebulized salbutamol in such patients significantly improves spirometry indices and relieves dyspnea more than single therapy without any side effect. But optimization of the drugs’ dose require to be designed in future studies.

## Provenance and peer review

Not commissioned, externally peer reviewed.

## Availability of data and materials

Are provided and applicable.

## Ethical approval

Study was approved by Kashan University of medical sciences board ethics committee and ethic approval. http://ethics.research.ac.ir/IR.KAUMS.MEDNT.REC.1397.006.

## Authors’ contribution

1-Abdoulhossein Davoodabadi: Investigation, Conceptualization, Methodology, Supervision, Writing – original draft.

2-Mohammadali Saba: Investigation, Conceptualization, Methodology, Supervision.

3-Azin Ghaffari: Investigation, Conceptualization, data collection, Methodology Conceptualization, Methodology.

4-Hamidreza Gilasi: data analysis Investigation, Conceptualization.

5-Babak Haghpanah: Writing – original draft.

## Funding

There is no source of funding other than the authors.

## Consent

We obtain a written informed consent from all of the participants.

## Registration of research studies

Unique Identifying number or registration ID: WHO IRCT (Iranian Registry of Clinical trial number) = IRCT201707126187N5.

## Guarantor

Abdoulhossein Davoodabadi.

## Limitation of the study

In this study we had a well COPD patients that may did not resemble the general population, however we should consider that they were really the patients who did not have any additional dyspnea (due to exacerbating disease) but had some dyspnea in their daily activities, then by this study we assessed dyspnea which was sensed by patients in exertion every day.

## Declaration of competing interest

The authors declare no conflict of interest.
